# Children and adolescents at risk for seclusion and restraint in inpatient psychiatric treatment: a case control study

**DOI:** 10.1186/s13034-024-00791-3

**Published:** 2024-08-13

**Authors:** Klara Czernin, Anselm Bründlmayer, Anna Oster, Josef S. Baumgartner, Paul L. Plener

**Affiliations:** 1 Department of Child and Adolescent Psychiatry, Medical University of Vienna, Währinger Gürtel 18-20, 1090 Vienna, Austria; 2 Department of Psychiatry and Psychotherapy, Clinics in the Theodor-Wenzel-Werk, Berlin, Germany; 3 Clinical Division of Social Psychiatry, Department of Psychiatry and Psychotherapy, Medical University of Vienna, Vienna, Austria; 4 Department of Child and Adolescent Psychiatry and Psychotherapy, University of Ulm, Ulm, Germany

**Keywords:** Risk factors, Seclusion, Restraint, Child and adolescent psychiatry, Borderline personality disorder, Post-traumatic stress disorder

## Abstract

To reduce coercion in acute inpatient child and adolescent psychiatric units, a better understanding of individuals at risk for seclusion and/or restraint (S/R) is needed. We report data on the proportion of patients secluded/restrained and factors associated with higher risk of S/R. Identifying preventative mechanisms through risk stratification upon inpatient admission can aid the training of mental health professionals, and support shaping specific workflows for at-risk populations for example by joint crisis plans or post-coercion review sessions. Methods: A case-control study included all admissions (*n* = 782) to a department of child and adolescent psychiatry within 36 months between 2019 and 2022. Data on age, sex, out of home care, primary and comorbid ICD-10 diagnoses, length of stay, prior/multiple admissions were compared between admissions with and without S/R using chi square tests for categorical and t-tests for continuous variables. Uni- and multivariate binary logistic regression models were computed. Results: The overall proportion of S/R was 12.8% (*n* = 100). Females (*p* = 0.001), patients in out of home care (*p* < 0.001), with prior admission (*p* < 0.001), Post-traumatic stress disorder (PTSD; *p* < 0.001) and Borderline personality disorder (BPD; *p* < 0.001) were at a significantly higher risk of S/R. Length of stay in days (OR 1.01), out of home care (OR 3.85), PTSD (OR 6.20), BPD (OR 15.17), Attention deficit hyperactivity disorder (ADHD)/conduct disorder (OR 4.29), and manic episode/bipolar disorder (OR 36.41) were significantly associated with S/R in multivariate regression. Conclusions: Child and adolescent psychiatric staff should consider risk factors when using coercive measures. Patients with PTSD and/or BPD are the most vulnerable subgroups. Training of professionals and clinical practice need to be adapted in order to prevent the use of S/R and its potential hazards.

## Background

In child and adolescent psychiatric units, mechanical coercive measures such as seclusions and/or restraints (S/R) are used as a last resort to prevent patients from harming themselves or others. Since the use of S/R is potentially harmful to both patients and staff, the overarching goal is to reduce them to a minimum. Risks to patients include injury [1, 2], re-experiencing from past traumatic events [3], and the risk of developing symptoms of post-traumatic stress disorder (PTSD) [2]. Chieze et al. (2019) found rates of PTSD after S/R ranging from 25 to 47% in adult populations [2]. In retrospect, affected patients seem to perceive seclusions as less restrictive than restraints, and more frequently consider seclusions as justified. Some patients reported at least some helpful effects, for example calming them down [4]. Exploring service users’ experiences and needs is relevant for finding alternatives to S/R and improving practices [5]. The incidence of violence is a result of the complex interactions between patients and staff and is influenced by the culture of the unit [6]. Consequently, the use of S/R is expected to be closely related to this interplay. The use of S/R poses ethical and legal challenges for professionals, especially in the field of care for minors.

Studies report varying and sometimes high prevalence rates of coercive measure use in psychiatric facilities for children and adolescents [7, 8]. A review published in 2022 reported that the prevalence of S/R among inpatients in child and adolescent psychiatric units ranged from 6.5 to 29% [9].

Attempts to effectively reduce the use of S/R focus on structural levels, i.e., mental health service planning and evaluation [10], as well as on institutional levels with treatment concepts and staff training [11, 12]. A better understanding of affected underage patients is needed in order to minimize coercion on the patient level. Previous studies found a skewed distribution, with a small proportion of adolescents subjected to coercive measures repeatedly, while another proportion was affected only once or twice [13]. Data identifying clinical and sociodemographic risk factors for coercion are particularly relevant because they point to potential targets for prevention. Previous studies, including the development of machine-learning based algorithms [14], have yielded heterogeneous results, applied a wide range of methods and named a wide spectrum of risk factors [8, 13, 15–27].

Female sex has been associated with a higher risk of S/R in Finnish nationwide studies [8, 13]. This is further supported by a systematic review of European studies describing female adolescents as vulnerable [20]. Non-European studies tend to report younger age [18, 21, 22] and male sex as risk factors [20, 27]. A single study described an increase of risk with age in boys [23].

Several studies point to social perspective, with poorer family functioning associated with S/R [20], with foster care [16] and out of home placement [24] as further risk factors consolidating the link. Belonging to a minority group in predominantly White communities increased risk for S/R in some US studies [21, 23, 25, 27]. However, in sight of the sparsity of evidence, the complex interaction of children and adolescents at risk for S/R with their surroundings is least understood in social domains.

In contrast, more studies have focused on mental health characteristics of youth affected by S/R. Poorer psychosocial functioning associated with a mental disorder poses a risk for repeated S/R [13], and displaying more severe psychopathology is a risk factor for experiencing coercion [22]. Attention deficit hyperactivity disorder (ADHD) and conduct disorders (ICD-10 ‘F9’ category) [20], schizophrenia and mood disorders [8], and PTSD [19] are diagnostic categories linked to an increased risk of S/R. In respect of potentially traumatizing effects of S/R [2], it is further remarkable that children’s history of physical abuse is associated with the rate of S/R [19], and many adult psychiatric inpatients affected by coercive measures retrospectively report being abused in their childhood [28].

Lastly, aggression and/or violent behavior on the patient’s side are reported as important factors leading up to the use of S/R [22–24, 26], while higher counts of prior admissions and longer durations of admissions render the individual more prone to being affected by coercive measures [13, 15–18, 24, 27].

This paper presents data on the use of S/R in an acute psychiatric facility for children and adolescents in the city of Vienna, a metropolitan region in Austria.

The purpose of this study was to examine which of the adolescents’ sociodemographic or clinical characteristics influence the risk of S/R. In sight of heterogeneous previous research and conflicting arguments especially on the role of sex and age, this manuscript seeks to clarify and to confirm. A further aim was to identify individuals at risk, in order to shape necessary interventions and adapt clinical practice accordingly. Findings from this study could be used for risk stratification upon admission, and for creating new workflows for monitoring at-risk populations.

## Methods

### Setting and survey period

We performed a case-control study at the Department of Child and Adolescent Psychiatry at the Medical University of Vienna. All admissions to two psychiatric wards during a survey period of three years from April 1st, 2019 to April 30th, 2022 were included. The whole month of October 2020 was excluded due to relocation of facilities. The department of Child and Adolescent Psychiatry serves a geographically defined catchment area hosting 45% of Austria´s capital’s population under the age of 18 years, which includes approximately 155,000 individuals. Treatment is offered for all psychiatric disorders with one ward including a locked safety facility with four beds giving special considerations to psychiatric emergencies with acute endangerment. S/R are predominantly used in the locked ward, but not exclusively. Maximum combined capacity of both wards was between 28 and 30 beds in the study period.

### Data collection

We collected data on all in-patient admissions and coercive measures in the 36 months of the survey period from electronic medical records. Clinical variables and the incidence of S/R was gathered on case level. One patient who was admitted 19 times (extensive number of multiple admissions) was excluded from the analysis based on authors’ consensus, presenting as a statistical outlier.

### Definition of mechanical coercive measures in the Austrian Mental Health Care Act

The Austrian civil commitment law (“*Unterbringungsgesetz*”) regulates the practice of (mechanical) coercive measure use in psychiatric departments in Austria. The following types of coercive measures are used in Austrian psychiatric wards: mechanical restraint, which restricts the patient´s freedom of movement, including mechanical devices; seclusion, which consists of involuntarily confining the patient in a closed room; physical restraint, in which the patient is physically restrained by staff; and pharmacological restraint, in which a patient is given medication with sedating characteristics. In this study, we did not consider physical restraint and pharmacological restraint due to limited comparability of data between time-points following a structural change in documentation.

### Sociodemographic, clinical variables and coercive measures

Sociodemographic variables included: sex (female/male), age in years and out of home care (yes/no). Clinical variables included: length of stay in days, prior admission in survey period (yes/no), total number of admissions, involuntary admission (yes/no), main and comorbid psychiatric diagnoses established by board certified consultants in child and adolescent psychiatry according to ICD-10: total number of psychiatric diagnoses, main diagnoses as dichotomous variables for schizophrenia spectrum disorder, PTSD, personality disorder (PD), ADHD or conduct disorder, manic episode or bipolar disorder, substance use disorder, depressive disorder, anxiety disorder; psychiatric comorbidity (more than one psychiatric diagnosis, yes/no), comorbid diagnoses as dichotomous variables for PTSD, PD, substance use disorder, or intellectual disability, and any presence of PTSD (yes/no) or PD diagnosis (yes/no).

Event of any mechanical coercive measures (seclusion and/or restraint, yes/no) was retrieved from the legal records, measures of S/R have to be reported immediately to the responsible court following the Austrian Mental Health Care Act. For patients with more than one admission within the study period, information on comorbid psychiatric diagnoses and out of home care was collected from the chronologically latest admission.

### Statistical analysis

Descriptive statistics were performed using frequencies and percentages of the respective study group for categorical variables and means and standard deviations (SD) for continuous variables. Chi squared tests for categorical variables, and Levene’s F test and Student’s t tests for continuous variables were used to assess differences between groups with and without S/R. Univariate and multivariate binary logistic regression analyses (regression coefficient B, odds ratios (OR) with 95% confidence intervals (CI)) were applied to analyze the impact of sex, age, out of home care, length of admission, prior admission, and main and comorbid psychiatric diagnoses (independent variables) on the occurrence of S/R (dependent variable). A p value < 0.05 was regarded as statistically significant. Bonferroni correction was applied in order to adjust for multiple testing in bivariate associations and univariate regression analyses. All statistical analyses were conducted using IBM SPSS Statistics version 29 for Apple Macintosh OSX. The study received a positive vote from the Ethics Committee of the Medical University of Vienna (reference no.: 1639/2019).

## Results

### Study sample

The sample comprised 782 cases. The mean age was 14.7 years (SD 2.0), with a range from six to 18 years. 604 (77.2%) were female. The mean length of stay was 29.5 days (SD 37.8), ranging from one to 319 days. In 478 (61.1%) cases, the adolescents had previous hospitalizations, and psychiatric comorbidity was frequent (> one ICD-10 diagnosis in 56.3% of admissions). When PD was diagnosed as a main diagnosis (*n* = 112) or comorbid diagnosis (*n* = 77), it was predominantly a diagnosis of Borderline PD (BPD, *n* = 108 main diagnosis; *n* = 72 comorbid diagnosis). Proportion of involuntary admission was 27.5% (217 cases). In 265 cases (33.9% of all admissions), patients had been in an out of home placement. Comparisons of sociodemographic and clinical characteristics of the two groups are listed in detail in Table 1.

**Table 1:** Bivariate associations (chi-squared tests) and comparisons of means (t tests) between sociodemographic and clinical characteristics of the sample and occurrence of S/R (*n* = 782 admissions); Bonferroni adjusted *p* < 0.002 (0.05/23) was considered significant

	Affected by S/R (*n* = 100)	Non-affected by S/R (*n* = 682)	χ^2^	T (df)	*p*
Age, mean (SD)	14.9 (1.8)	14.6 (2.0)	n.a.	1.285 (780)	0.199
Female sex, n (%)	90 (90%)	514 (75.4%)	10.622	n.a.	0.001
Length of stay in days, mean (SD)	30.6 (56.0)	29.4 (34.4)	n.a.	0.220 (110.182)	0.827
Prior admission, n (%)	82 (82%)	396 (58.1%)	21.027	n.a.	< 0.001
Total number of admissions, mean (SD)	11.6 (10.1)	3.2 (4.2)	n.a.	8.276 (103.939)	< 0.001
Involuntary admission, n (%)	100 (100%)	115 (16.9%)	302.390	n.a.	< 0.001
Out of home care, n (%)	71 (71%)	194 (28.4%)	70.492	n.a.	< 0.001
Psychiatric comorbidity, n (%)	67 (67%)	373 (54.7%)	5.369	n.a.	0.020
Total number of diagnoses, mean (SD)	2.00 (1.06)	1.81 (0.95)	n.a.	1.860 (780)	0.063
Primary diagnosis of					
Schizophrenia spectrum disorder, n (%)	2 (2%)	40 (5.9%)	2.563	n.a.	0.109
PTSD, n (%)	32 (32%)	98 (14.4%)	19.558	n.a.	< 0.001
PD, n (%)	42 (42%)	70 (10.3%)	71.582	n.a.	< 0.001
ADHD or conduct disorder, n (%)	7 (7%)	45 (6.6%)	0.023	n.a.	0.880
Manic episode or bipolar disorder, n (%)	2 (2%)	4 (0.6%)	2.289	n.a.	0.130
Substance use disorder, n (%)	0 (0%)	10 (1.5%)	1.485	n.a.	0.223
Depressive disorder, n (%)	6 (6%)	143 (21.0%)	12.668	n.a.	< 0.001
Anxiety disorder, n (%)	1 (1%)	18 (2.6%)	0.989	n.a.	0.320
Comorbid diagnosis of					
PTSD, n (%)	13 (13%)	64 (9.4%)	1.284	n.a.	0.257
PD, n (%)	33 (33%)	44 (6.5%)	69.245	n.a.	< 0.001
Substance use disorder, n (%)	2 (2%)	22 (3.2%)	0.441	n.a.	0.507
Intellectual disability, n (%)	2 (2%)	10 (1.5%)	0.164	n.a.	0.685
Any diagnosis of					
PTSD, n (%)	43 (43%)	144 (21.1%)	22.959	n.a.	< 0.001
PD, n (%)	66 (66%)	104 (15.2%)	132.031	n.a.	< 0.001

ADHD = attention deficit hyperactivity disorder; PD = personality disorder; PTSD = post-traumatic stress disorder; S/R = seclusion and/or restraint

n.a.=not applicable

### Risk factors for S/R

Of the 782 cases admitted between April 2019 and April 2022, 100 (12.8%) experienced S/R. 19 cases (2.4%) experienced seclusion and 86 cases experienced mechanical restraint (11.0%). Cases affected by S/R were significantly more often of female sex, were more frequently admitted previously and had higher numbers of total admissions. Psychiatric comorbidity was considerably higher in the S/R group, as well as being in out of home care.

Having a primary diagnosis of schizophrenia spectrum disorder, ADHD or conduct disorder, manic episode or bipolar disorder, substance use disorder or anxiety disorder was not associated with differences in S/R rate. Depressive disorder as a primary diagnosis was significantly more frequent in the no-S/R group. Two diagnostic entities were statistically most linked to the rate of S/R: PTSD as a primary diagnosis and any diagnosis of PTSD had higher rates of S/R. A diagnosis of PD as primary, comorbid, or any diagnosis was significantly associated with a higher rate of S/R.

### Univariate and multivariate regression models

The results of the univariate and multivariate binary regression analyses are presented in Table 2. The univariate analyses showed that female sex, prior admission, out of home care, main diagnoses of PTSD and PD, as well as a comorbid diagnosis of PD were associated with a higher rate of S/R, whereas a main diagnosis of depressive disorder was associated with a lower rate.

To ascertain the independent effects of single items on the likelihood of S/R, we joined the predictors in a multivariate logistic regression model. The model was statistically significant (χ^2^(18) = 168.466; *p* < 0.001) and explained 36.3% (Nagelkerke R^2^) of the variance and correctly classified 87.9% of cases. Sex did not significantly contribute to the model. Cases in out of home care were 3.67 times more likely to be affected than those not in out of home care. Regarding diagnoses, PTSD, PD, ADHD or conduct disorder, and manic episode or bipolar disorder significantly added to the model and were associated with higher likelihoods of S/R. Longer admissions were also associated with the occurrence of S/R. Age, prior admission, psychiatric comorbidity and remaining diagnostic categories did not significantly contribute to the multivariate model.

**Table 2:** Correlates of S/R use among 782 children and adolescents between April 2019 and April 2022

	Univariate model (*p* < 0.0028)			Multivariate model (*p* < 0.05)		
Variable	B	OR (p)	95% CI	B	OR (p)	95% CI
Age	0.073	1.08 (0.199)	0.96 − 1.20	0.011	1.01 (0.894)	0.86-1.19
Female sex	1.079	2.94 (0.002)	1.50–5.78	0.646	1.91 (0.119)	0.85-4.29
Length of stay	0.001	1.00 (0.755)	1.00–1.00	0.009	1.01 (0.004)	1.00-1.02
Prior admission	1.191	3.29 (< 0.001)	1.93–5.60	0.282	1.33 (0.407)	0.68-2.58
Out of home care	1.818	6.16 (< 0.001)	3.88–9.78	1.299	3.67 (< 0.001)	2.03–6.62
Psychiatric comorbidity	0.520	1.68 (0.021)	1.08–2.62	0.039	1.04 (0.907)	0.54 − 2.00
Primary diagnosis of						
Schizophrenia spectrum disorder	−1.116	0.33 (0.128)	0.08-1.38	0.529	1.70 (0.529)	0.33-8.78
PTSD	1.031	2.80 (< 0.001)	1.75–4.49	1.796	6.02 (< 0.001)	2.50-14.55
PD	1.845	6.33 (< 0.001)	3.97–10.11	2.727	15.29 (< 0.001)	6.17–37.90
ADHD or conduct disorder	0.063	1.07 (0.880)	0.47-2.43	1.499	4.48 (0.014)	1.35–14.89
Manic episode or bipolar disorder	1.241	3.46 (0.155)	0.63-19.14	3.858	47.38 (< 0.001)	6.54-343.04
Substance use disorder	−19.298	0.00 (0.999)	0–0	-16.980	0.00 (0.999)	0–0
Depressive disorder	−1.425	0.24 (< 0.001)	0.10-0.56	0.562	1.76 (0.328)	0.57-5.42
Anxiety disorder	− 0.987	0.37 (0.339)	0.05-2.82	0.581	1.79 (0.615)	0.19-17.16
Comorbid diagnosis of						
PTSD	0.367	1.44 (0.259)	0.76-2.73	0.001	1.00 (0.999)	0.44-2.29
PD	1.966	7.14 (< 0.001)	4.26–11.97	0.998	2.71 (0.007)	1.31–5.62
Substance use disorder	− 0.491	0.61 (0.511)	0.14-2.64	0.026	1.03 (0.978)	0.17-6.26
Intellectual disability	0.316	1.37 (0.686)	0.30-6.35	0.774	2.17 (0.403)	0.35-13.30

ADHD = attention deficit hyperactivity disorder; CI = confidence interval; OR = odds ratio; PD = personality disorder; PTSD = post-traumatic stress disorder; S/R = seclusion and/or restraint

## Discussion

This study is the first to report risk factors for S/R in children and adolescents in Austria. It contributes to the limited knowledge about how young patients in inpatient settings are differentially affected by the use of S/R. Previous research on this topic has yielded inconsistent results and sometimes contradictory findings regarding demographic, social and clinical influencing factors [8, 13, 15–27]. Consequently, our study could assist in improving clinical practice and de-escalation strategies in child and adolescent psychiatry.

The sample investigated is representative for service users in a central European urban region, as the department has a regionalized supply mandate, meaning that all individuals requiring psychiatric treatment in a defined catchment area are referred. The study sample was predominantly female, and in mid- or late adolescence, 87.1% of patients had passed their 13th birthday. Most frequent reasons for referrals and admissions are worsening of symptoms in the context of depressive disorders, PTSD, personality disorders, ADHD and conduct disorders, as well as crises with psychological implications in the context of violence, trauma, and dysfunctional care systems. Around 13% of all inpatients in our sample experienced S/R, most frequently due to severe auto-aggression. This rate seems comparable to an Irish child and adolescent psychiatric sample assessed between 2018 and 2021 in which 6% experienced seclusion and 18% physical restraint [29].

### Demographic characteristics

Biological age is heterogeneously linked to risk for S/R across the globe [20]. Older age was a risk factor in European studies, whereas younger patients were at risk in US American and Australian studies [18, 21, 22]. While patients affected by S/R in our sample were slightly older than average, the difference lacked statistical significance. European teenagers seem to be at highest risk for S/R during late adolescence. However, age alone doesn’t seem to independently predict risk for S/R. Differences in organization of mental health care for children and adolescents between geographical regions could be a reason for the inconsistent results worldwide.

Females were more likely to be affected by S/R in our sample, which is consistent with previous Scandinavian studies [8, 13] and other European countries [20]. However, sex did not predict proportion of S/R in our sample when controlling for other influencing factors in the multivariate regression model. Moreover, in analogy to age as a risk factor, there is inconsistency in the research when considering findings from across the world [20, 23]. Structural and legislative implications, as well as the role of psychiatry in the respective society might contribute to different findings. Nevertheless, our study underlines the at-risk for S/R status of females in European child and adolescent psychiatric systems, and the need for preventive action.

### Social characteristics

Out of home placement is a stable risk factor for being affected by S/R across various studies, which is also confirmed in our study [16, 24]. This consistency is further supported by poorer family functioning as a risk factor [22]. Reasons for this vulnerability may include the presence of more pronounced psychosocial problems. Children in out of home care have a high prevalence of psychiatric disorders, more severe psychopathology, and a higher prevalence of trauma-related disorders and cognitive processes [30, 31]. When admitting patients from out of home care settings and unstable family and social contexts, clinicians should be aware of the higher risk for S/R and adapt treatment planning accordingly.

### Mental health characteristics

A diagnosis of BPD or any other PD in adolescence was not previously reported as a risk factor for S/R, however, it was associated with a higher rate in our sample. One explanation for this novelty might be recent changes in diagnostic recommendations in DSM-5 and ICD-11 [32, 33] and the latest German clinical guideline following suit and recommending diagnosis from the age of 12 [34]. Until a decade ago, clinicians were reluctant to diagnose PD in adolescents [35, 36]. Recently, there has been a shift towards screening for personality organization deficits in adolescents in order to avoid delayed treatment adaptations [37]. Complex and severe psychopathological features after traumatic experiences are also increasingly diagnosed as changes in personality, which might add to higher prevalence of PD in modern samples [38, 39]. However, it is unlikely that a change in diagnostic practice alone will have such a marked effect on risk for S/R. Especially patients with severe PD and impulsivity can exhibit serious acts of auto-aggressive behavior due to which, when all other de-escalation measures fail, S/R are applied to protect the patient. Severity of psychopathology increases the risk for S/R [22]. Edlinger et al. (2014) reported that adult inpatients with a cluster B PD and with organic neurocognitive disorders were more at risk of restraint than patients with schizophrenia in a locked unit [40]. Implementation of a clinical management guideline for PDs was shown to drastically reduce the use of restraint in adult inpatients with BPD [41]. Implementing aspects of dialectical behavioral therapy (DBT) into inpatient treatment settings is helpful in responding to suicidal behavior and self-harm [42]. DBT-informed interventions might be valuable in psychiatric practice in order to deescalate and find alternatives to S/R.

PTSD has been named as a risk factor for S/R in a pediatric psychiatry day hospital [19], and was also predictive for a higher rate of S/R in this study. History of physical abuse and childhood abuse are frequent in adolescent and adult inpatients experiencing S/R [19, 28]. As a potential hazard, S/R in psychiatric practice carries the risk of traumatizing the individuals involved, on top of re-traumatizing potentials of coercion in traumatized patients [2]. Standardized post-coercion review sessions have been shown to reduce PTSD symptoms in adults and may also be of relevance in adolescence [43]. It appears that some psychiatric treatments therefore risk further burdening those with comorbid or complicating mental health issues. Based on clinical experience, and substantiated by our findings, especially patients with comorbid PTSD and BPD constitute a highly vulnerable sub-group of adolescent psychiatric inpatients. For these individuals, inpatient treatment in a psychiatric unit often presents the only option for being taken care of, considering a lack of outpatient treatment services and overload of caring institutions or families. Besides trauma informed care, adapted treatment strategies for the care for these vulnerable patients are needed and furthermore underline the notion that patients with BPD should primarily be treated in an outpatient setting [34].

In line with previous literature, patients with manic episodes and bipolar disorder [8] and ADHD or conduct disorder [20] were at a higher risk for S/R, but much less affected than those with PTSD and BPD. Adolescents with early onsets of schizophrenia spectrum disorder did not show elevated risk for S/R in our sample.

### Treatment characteristics

In the present study, prior and longer admissions were significant risk factors for S/R. These factors were also consistently associated with S/R in previous studies [13, 15–18, 24, 27]. Longer hospitalizations occur with severe psychopathology, multiple psychiatric diagnoses, and medical comorbidities [44]. Higher rates of rehospitalization in a child and adolescent psychiatric unit were linked to history of trauma and active bullying [45].

### Strengths and limitations

A methodological strength of our study is the relatively long study period of 3 years and the representativeness of the sample due to the geographical catchment area. This means that all underaged patients living in certain districts of Vienna are referred to this department. We had access to data on all registered S/R episodes, as these must be meticulously collected and documented by law. Case-level analysis allows for high clinical validity and everyday applicability, as the identified risk factors may serve as ‘red flags’ in any patient admission process. The robust statistical results which are greatly consistent with prior studies, confirm the validity of the highly relevant clinical perceptions.

A limitation of this study is the monocentric design. Some variables had low counts, which limits generalizability. Physical restraint and pharmacological restraint were not included due to limited comparability of data. It is further possible that some controls experienced S/R at other institutions, for example if they moved in or out of the catchment area. Patients might have been admitted multiple times before or after the study interval, and readmissions might have influenced results to a stronger degree, as each readmission was counted as a new case. On top of that, the COVID-19 pandemic and lockdown measures might have acted as a confounding variable, as the rate of S/R was highest in the first pandemic year of 2020 (16%), with 2021 (14%), 2019 (10%) and 2022 (5%) following. The method that some data on characteristics were taken from the chronologically latest admission clouds the clinical reality that several risk factors may be unknown at the time the at-risk individual is first admitted.

## Conclusion

Risk for S/R during child and adolescent inpatient psychiatric treatment remains common. Moreover, patients diagnosed with BPD have been newly identified as a high-risk group, and to date there are no disorder-specific approaches for the prevention of coercion in this patient cohort. Frequent co-occurrence of PD and PTSD in the group of coercive measures hints towards valuable information to improve clinical practice.

Firstly, training of mental health staff should raise awareness that adolescent patients with a PD as well as those with PTSD are at-risk groups for S/R. Structured and standardized risk stratification at time of admission may identify vulnerable individuals. Secondly, clinical management plans and de-escalation strategies need to be adapted, integrating elements of recovery-oriented and trauma-informed care as well as consider specific needs of young patients with PD. Lastly, asking for subjective experiences and preferences of patients regarding S/R use could be part of post-coercion review sessions as well as treatment planning at admission.

## Data Availability

The datasets generated and analyzed during the current study are available from the corresponding author on reasonable request.

## Abbreviations

S/R: Seclusion and/or restraint
PTSD: Post-traumatic stress disorder
BPD: Borderline personality disorder
ADHD: Attention deficit hyperactivity disorder
PD: Personality disorder
SD: Standard deviation
OR: Odds ratio
CI: Confidence interval
